# Detection of Merkel cell polyomavirus in multiple primary oral squamous cell carcinomas

**DOI:** 10.1007/s10266-023-00807-y

**Published:** 2023-03-25

**Authors:** Naoya Kitamura, Yumiko Hashida, Tomonori Higuchi, Seiji Ohno, Shinya Sento, Eri Sasabe, Ichiro Murakami, Tetsuya Yamamoto, Masanori Daibata

**Affiliations:** 1 Department of Oral and Maxillofacial Surgery, Kochi Medical School, Kochi University, Nankoku, Kochi 783-8505 Japan; 2 Department of Microbiology and Infection, Kochi Medical School, Kochi University, Nankoku, Kochi 783-8505 Japan; 3 Department of Pathology, Kochi Medical School, Kochi University, Nankoku, Kochi 783-8505 Japan

**Keywords:** Oral squamous cell carcinoma, Oral microbiome, Merkel cell polyomavirus, Multiple primary oral cancers, Japanese

## Abstract

Oral microbiome studies have mainly focussed on bacteria, with the relationship between viruses and oral cancers remaining poorly understood. Oral cancers can develop even in the absence of any history of daily smoking or drinking. Oral cancer patients frequently have multiple primary cancers in the oral cavity and other organs, such as the upper gastrointestinal tract. Merkel cell polyomavirus (MCPyV) is a novel oncovirus identified from a subtype of skin cancer in 2008. In this study, we investigated the potential involvement of MCPyV in the pathogenesis of oral squamous cell carcinoma (OSCC). Participants comprised 115 Japanese patients with OSCC (single primary: 109 tumours in 109 patients; multiple primaries: 16 tumours in 6 patients) treated in our department between 2014 and 2017. DNA was extracted from formalin-fixed paraffin-embedded specimens of primary lesions. MCPyV DNA copy counts were analysed by quantitative real-time polymerase chain reaction. Twenty-four of the 115 patients (20.9%) were positive for MCPyV DNA. No association was found between presence or absence of MCPyV DNA and clinical characteristics other than number of primary lesions. The MCPyV DNA-positive rate was significantly higher for multiple primary OSCCs (62.5%, 10/16 tumours) than for single primary OSCCs (16.5%, 18/109 tumours; *P* < 0.001). Furthermore, MCPyV DNA load was significantly higher for patients with multiple primaries (*P* < 0.05). MCPyV was observed more frequently and DNA load was significantly higher with multiple primary OSCCs than with single primary OSCC. MCPyV may play some role as an oncovirus for multiple primary OSCCs.

## Introduction

The gut microbiome is widely recognised as being involved in the pathogenesis and development of various cancers [1, 2]. Recently, periodontal pathogenic bacteria such as *Fusobacterium nucleatum* and *Aggregatibacter actinomycetemcomitans* have been reported to be involved in the pathogenicity of colorectal cancer and/or oesophageal cancer [3, 4], and we have also suggested that these bacteria are involved in oral squamous cell carcinoma (OSCC) [5]. Oral microbiome studies have mainly focussed on bacteria, and the relationship between viruses and oral cancers is poorly understood [6]. Regarding the association between oral and pharyngeal diseases and viruses, associations have been identified between hepatitis C virus and oral lichen planus, between human papillomavirus (HPV) and oropharyngeal cancer, and between Epstein-Barr virus and both nasopharyngeal cancer and methotrexate-associated lymphoproliferative disorders. However, the relationship between oral latent viruses and OSCC remains unclear.

Smoking and alcohol drinking are listed as risk factors for the development of head and neck cancers, including oral cancer, and are also risk factors for cancers of other organs. On the other hand, many cases involve the development of oral cancers in the absence of any history of daily smoking or drinking, and other risk factors besides smoking and drinking may thus be involved. Oral cancer patients frequently have multiple primary cancers in the oral cavity and other organs, such as the upper gastrointestinal tract and lungs [7–11]. Such phenomena are explained by the concept of field cancerization and cell competition, in which various gene mutations and epigenetic abnormalities accumulate in tissues and organs that have been exposed to cancer-inducing factors over a prolonged period [12–16].

Merkel cell polyomavirus (MCPyV) is a novel oncovirus identified from a subtype of skin cancers in 2008. This virus is often present as the wild type in the oral cavity [17]. Furthermore, human polyomavirus type 6 (HPyV6) and type 7 (HPyV7) were discovered in 2010 as cutaneous polyomaviruses that are also associated with some tumours, suggesting aspects of oncoviruses [18]. To investigate the potential involvement of virus groups in OSCC, we focussed on MCPyV as a human polyomavirus.

## Materials and methods

### Patients

We recruited 115 Japanese patients (single primary: 109 tumours in 109 patients; multiple primaries: 16 tumours in 6 patients) with OSCC treated in the Department of Oral and Maxillofacial Surgery at Kochi Medical School Hospital during the 4-year period from 2014 to 2017. We retrospectively examined the following clinical information of the enrolled patients based on the medical records: sex, age, smoking/alcohol drinking history, primary site, TNM classification (according to the 8^th^ edition of the AJCC/UICC TNM classification), clinical stage, histopathological differentiation, and prognosis (follow-up period: 60 months). Multiple oral cancers were defined in accordance with the second edition of the general rules for clinical and pathological studies on oral cancer [19], as the occurrence of two or more primary cancers fulfilling the following conditions: (1) cancer located at different sites according to the UICC classification; (2) cancer located at the same but contralateral sites; (3) cancer located at ipsilateral sites, but discontinuously and clinically separated by ≥ 2.0 cm; or (4) each lesion histopathologically confirmed to represent carcinoma. All study protocols were approved by the ethics committee of Kochi Medical School, Kochi University Hospital.

### Detection and evaluation of MCPyV DNA viral load in OSCCs

Using formalin-fixed paraffin-embedded (FFPE) specimens of the primary lesion, DNA was extracted from three 10-μm-thick slices of tumour using a WaxFree DNA Extraction Kit (TrimGen; Sparks, Maryland, USA). Microtome blades were changed between each embedded block to prevent cross-contamination between specimens. Next, using 200 ng of extracted DNA, MCPyV DNA copy counts were analysed by quantitative real-time polymerase chain reaction (qPCR) (TaqMan Gene Expression Assays; Applied Biosystems™, Waltham, Massachusetts, USA). The protocols of reaction conditions were 50 °C for 2 min and 95 °C for 10 min, followed by 50 cycles of 95 °C for 15 s and 60 °C for 1 min. For MCPyV PCR, the target viral gene was the ST gene (primers and probe sequences are shown below) [20]. All qPCR was performed in triplicate, with a result of amplified products in ≥ 2 samples representing a MCPyV DNA-positive result. *RNase P* gene (primers and probe sequences shown below) was used as an internal control.

[MCPyV ST gene-targeted primers and probe sequences].

Forward: GCAAAAAAACTGTCTGACGTGG.

Reverse: CCACCAGTCAAAACTTTCCCA.

Probe: FAM-TATCAGTGCTTTATTCTTTGGTTTGGATTTC-TAMRA.

[*RNase P* primers and probe sequences].

Forward: AGATTTGGACCTGCGAGCG.

Reverse: GAGCGGCTGTCTCCACAAGT.

Probe: FAM-TTCTGACCTGAAGGCTCTGCGCG-TAMRA.

To calculate viral copy numbers, the polymerase chain reaction (PCR) product was cloned into the pMD20-T vector (Takara Bio, Japan), and sixfold serial dilutions of the cloned plasmid DNA were used to generate a standard curve. Results are expressed as viral copies per nanogram of DNA and viral copies per cell. *RNase P* was used as a housekeeping gene to control the quality of DNA and PCR reactions [20–22]. *RNase P* exists as a single-copy gene per haploid genome (2 copies per human cell); we can therefore calculate viral copies per cell when we quantify gene copy numbers.

### Association between MCPyV DNA and clinical characteristics

These MCPyV statuses were retrospectively compared with the clinical characteristics of the patient (e.g. sex, age, smoking/alcohol drinking history, primary site, TNM classification, clinical stage, histopathological differentiation, and prognosis).

### Statistical analysis

Overall survival (OS) and disease-specific survival (DSS) were calculated using the Kaplan–Meier method and compared using the log-rank test. For categorical data analysis of MCPyV status and clinical characteristics among patient groups stratified by single or multiple OSCC, Fisher's exact test was used. Comparisons of mean age were assessed using Student's *t* test, and comparisons of MCPyV DNA viral loads were assessed using the Mann–Whitney *U* test. Values of *P* < 0.05 were considered statistically significant. EZR (version 1.55; Saitama Medical Center, Jichi Medical University, Saitama, Japan. See https://www.jichi.ac.jp/saitama-sct/SaitamaHP.files/statmedEN.html) was used for all statistical analyses [23], and the statistical methodology was reviewed by a statistician at Kochi Medical University.

## Results

### Clinical characteristics and sample preparation

The clinical information of patients is shown in Table 1. Most patients were male (64 patients, 55.7%) and the mean age was 71.9 ± 12.9 years (range 37–97 years). In terms of smoking history, Brinkman index was low (< 400) in 67 patients (61.5%), and 65 patients (60.2%) had no drinking history. The most common primary site was the tongue (38.3%), followed by the mandibular gingiva (27.0%) and the maxillary gingiva (10.4%). The most common T classification was T2 (37.4%), the most common N classification was N0 (65.2%), and almost all cases were M0 (99.1%). In terms of clinical stage, stage IV (40%) was the most common, followed by stage I (26.1%), stage II (24.3%), and stage III (9.6%). The most common histological differentiation was well-differentiated (82.6%), and the vast majority of treatments were surgical (91.3%) (Table 1). Patients with multiple primary OSCCs comprised 5.2% of the population (6/115 patients). Regarding the distribution of multiple primary OSCCs, double cancers were seen in 3 patients, triple cancers in 2 patients, and quadruple cancers in 1 patient. We therefore prepared DNA from FFPE samples for 125 cases (single primary: 109 tumours; multiple primaries: 16 tumours).

**Table 1:** Clinicopathological characteristics of OSCC patients

	Total (*n* = 115)	
	*n*	%
Sex		
Male	64	55.7
Female	51	44.3
Age		
Mean ± SD (Range)	71.9 ± 12.9 (37–97)	
Smoking (Brinkman index)*		
0–400	67	61.5
401–1000	28	25.7
1001–4000	14	12.8
Alcohol consumption**		
None	65	60.2
A few times	7	6.5
Every day	36	33.3
Tumour site		
Tongue	44	38.3
Lower gingiva	31	27.0
Upper gingiva	12	10.4
Floor of the mouth	11	9.6
Cheek mucosa	8	7.0
Palate	6	5.2
Lip	3	2.6
T classification		
1	36	31.3
2	43	37.4
3	4	3.5
4	32	27.8
N classification		
0	75	65.2
1	12	10.4
2	25	21.7
3	3	2.6
M classification		
0	114	99.1
1	1	0.9
Stage		
I	30	26.1
II	28	24.3
III	11	9.6
IV	46	40.0
Differentiation		
Poor	4	3.5
Moderately	16	13.9
Well	95	82.6
Treatment		
Surgery	105	91.3
CRT	4	3.5
Palliative care	6	5.2
Single/multiple OSCC		
Single OSCC	109 [109]	94.8 [87.2]
Multiple OSCC	6 [16]	5.2 [12.8]
Double	3 [6]	2.6 [4.8]
Triple	2 [6]	1.3 [4.8]
Quadruple	1 [4]	1.3 [3.2]
Detection of MCPyV DNA		
Positive	24 [28]	20.9 [22.4]
Negative	91 [97]	79.1 [77.6]

[]: Tumour no

*n = 109

**n = 108

### Detection of MCPyV DNA viral load in OSCCs

Among the 115 patients, 24 patients (20.9%) showed positive results for MCPyV DNA (Table 1). No association was found between presence or absence of MCPyV DNA and sex, age, smoking history, alcohol drinking history, primary site, T and M classifications, clinical stage, histopathological differentiation, or treatment (Table 2). However, N classification differed significantly between the MCPyV-positive and -negative groups (*P* < 0.05, Fisher's exact test) (Table 2), and the MCPyV-positive rate was significantly higher in the multiple-OSCC group (62.5%, 10/16 tumours) than in the single-OSCC group (16.5%, 18/109 tumours; *P* < 0.001, Fisher's exact test) (Tables 2, 3).

**Table 2:** MCPyV status and clinicopathological characteristics of patients with OSCC

	Total (115 patients), [125 tumours]	MCPyV positive (24 patients), [28 tumours]		MCPyV negative (91 patients), [97 tumours]		*P* value
	*n*	*n*	%	*n*	%	
Sex						
Male	64	11	45.8	53	58.2	0.357
Female	51	13	54.2	38	41.8	
Age						
Mean ± SD (Range)	71.9 ± 12.9 (37–97)	71.5 ± 12.4 (37–88)		73.4 ± 14.8 (37–97)		0.529
Smoking (Brinkman index)*						
0–400	67	18	75.0	49	57.6	0.353
401–1000	28	4	16.7	24	28.2	
1001–4000	14	2	8.3	12	14.1	
Alcohol consumption**						
None	65	16	66.7	49	58.3	0.16
A few times	7	3	12.5	4	4.8	
Every day	36	5	20.8	31	36.9	
Tumour site						
Tongue	44 [49]	7 [9]	29.2 [32.1]	37 [40]	40.7 [41.2]	0.684 [0.602]
Lower gingiva	31 [33]	8	33.3 [28.6]	23 [25]	25.3 [25.8]	
Upper gingiva	12	3	12.5 [10.7]	9	9.9 [9.3]	
Floor of the mouth	11	1	4.2 [3.6]	10	11.0 [10.3]	
Cheek mucosa	8 [10]	2 [3]	8.3 [10.7]	6 [7]	6.6 [7.2]	
Palate	6 [7]	2 [3]	8.3 [10.7]	4	4.4 [4.1]	
Lip	3	1	4.2 [3.6]	2	2.2 [2.1]	
T classification						
1	36 [41]	8 [11]	33.3 [39.3]	28 [30]	30.8 [30.9]	0.344 [0.218]
2	43 [46]	12 [13]	50.0 [46.4]	31 [33]	34.1 [34.0]	
3	4 [5]	0	0	4 [5]	4.4 [5.2]	
4	32 [33]	4	16.7 [14.3]	28 [29]	30.8 [29.9]	
N classification						
0	75 [84]	16 [20]	66.7 [71.4]	59 [64]	64.8 [66.0]	**0.0324 [0.0489]**
1	12 [13]	6	25.0 [21.4]	6 [7]	6.6 [7.2]	
2	25	2	8.3 [7.2]	23	25.3 [23.7]	
3	3	0	0	3	3.3 [3.1]	
M classification						
0	114 [124]	24 [28]	100 [100]	90 [96]	98.9 [99.0]	1 [1]
1	1	0	0	1	1.1 [1.0]	
Stage						
I	30 [34]	7 [9]	29.2 [32.1]	23 [25]	25.3 [25.8]	0.061 [0.0585]
II	28 [31]	7 [9]	29.2 [32.1]	21 [22]	23.1 [22.7]	
III	11 [13]	5	20.8 [17.9]	6 [8]	6.6 [8.2]	
IV	46 [47]	5	20.8 [17.9]	41 [42]	45.1 [43.3]	
Differentiation						
Poor	4	1	4.2 [3.6]	3	3.3 [3.1]	0.792 [0.895]
Moderately	16	4	16.7 [14.3]	12	13.2 [12.4]	
Well	95 [105]	19 [23]	79.2 [82.1]	76 [82]	83.5 [84.5]	
Treatment						
Surgery	105 [114]	22 [26]	91.7 [92.9]	83 [88]	91.2 [90.7]	0.571 [0.442]
CRT	4 [5]	0	0	4 [5]	4.4 [5.2]	
Palliative care	6	2	8.3 [7.1]	4	4.4 [4.1]	
The number of OSCC						
Single OSCC	109	18	75.0 [64.3]	91	100 [93.8]	**0.0000 [0.0002]**
Multiple OSCC	6 [16]	6 [10]	25.0 [35.7]	0 [6]	0 [6.2]	

Statistically significant *P* values are in bold (*P* < 0.05)

*n = 109 (Single: 103 patients, Multiple: 6 patients)

**n = 108 (Single: 102 patients, Multiple: 6 patients)

**Table 3:** Comparison of clinicopathological characteristics for patients with single vs. multiple OSCCs

	Single OSCC (109 patients, 109 tumours)		Multiple OSCC (6 patients, 16 tumours)		*P* value
	n	(%)	n	(%)	
Sex					
Male	62	(56.9)	2	(33.3)	0.404
Female	47	(43.1)	4	(66.7)	
Age					
Mean ± SD (Range)	71.8 ± 12.6 (37–97)		74.7 ± 18.9 (37–87)		0.596
Smoking (Brinkman index)*					
0–400	61	(59.2)	6	(100)	0.195
401–1000	28	(27.2)	0	(0)	
1001–4000	14	(13.6)	0	(0)	
Alcohol consumption**					
None	60	(58.8)	5	(83.3)	0.614
A few times	7	(6.9)	0	(0)	
Every day	35	(34.3)	1	(16.7)	
Tumour site***					
Tongue + Floor of the mouth	55	(50.5)	5	(31.25)	**0.0467**
Lower + Upper gingiva	40	(36.7)	5	(31.25)	
Others (Cheek mucosa + Palate + Lip)	14	(12.8)	6	(37.5)	
T classification***					
1 + 2	73	(67.0)	14	(87.5)	0.145
3 + 4	36	(33.0)	2	(12.5)	
N classification***					
0	69	(63.3)	15	(93.75)	**0.0199**
1 + 2 + 3	40	(36.7)	1	(6.25)	
M classification***					
0	108	(99.1)	16	(100)	1
1	1	(0.9)	0	(0)	
Stage***					
I + II	52	(47.7)	13	(81.25)	**0.0152**
III + IV	57	(52.3)	3	(18.75)	
Differentiation***					
Poor	4	(3.7)	0	(0)	1
Moderately	14	(12.8)	2	(12.5)	
Well	91	(83.5)	14	(87.5)	
Treatment***					
Surgery	99	(94.3)	15	(93.75)	0.592
CRT	4	(3.7)	1	(6.25)	
Palliative care	6	(5.5)	0	(0)	
Detection of MCPyV DNA***					
Positive	18	(16.5)	10	(62.5)	**0.0002**
Negative	91	(83.5)	6	(37.5)	

Statistically significant *P* values are in bold (*P* < 0.05)

*n = 109 (Single: 103 patients, Multiple: 6 patients)

**n = 108 (Single: 102 patients, Multiple: 6 patients)

***Tumour No. (Total 125 tumours)

Further, comparisons between single- and multiple-OSCC groups revealed significant differences in primary tumour sites (Tongue and floor of the mouth, lower and upper gingiva, and Others), N classification (absence/presence of cervical lymph node metastases) and clinical stage (early/advanced stage) (*P* < 0.05 each, Fisher's exact test) (Table 3).

### Viral DNA load level of MCPyV-positive patients with multiple primary OSCCs vs. single primary OSCCs

**Fig. 1:**
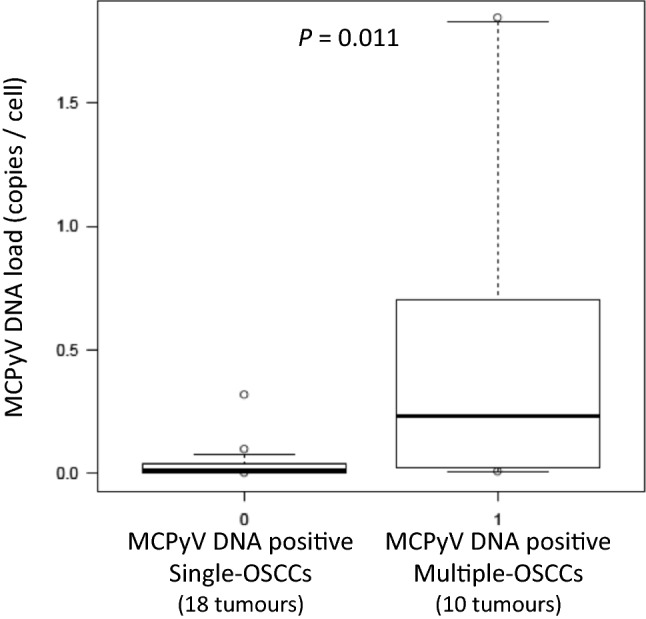
Distribution of MCPyV DNA load in patients with single vs. multiple OSCCs. Box plots showing comparisons between MCPyV DNA load in single and multiple OSCCs. In MCPyV DNA-positive single-OSCCs (18 tumours), median DNA load is 0.011 copies/cell (range 0.001–0.318 copies/cell; interquartile range 0.003–0.037 copies/cell). In MCPyV DNA-positive multiple-OSCCs (10 tumours), median DNA load is 0.23 copies/cell (range 0.007–1.842 copies/cell; interquartile range 0.023–0.67 copies/cell). MCPyV DNA loads are significantly higher in patients with multiple OSCCs than in patients with single OSCC (*P* = 0.011, Mann–Whitney *U* test)

Box plots in Fig. 1 show comparisons between MCPyV DNA loads in single and multiple OSCCs. In MCPyV DNA-positive single-OSCCs (18 tumours in 18 patients), median DNA load was 0.011 copies/cell (range 0.001–0.318 copies/cell; interquartile range 0.003–0.037 copies/cell). In MCPyV DNA-positive multiple-OSCCs (10 tumours in 6 patients), median DNA load was 0.23 copies/cell (range, 0.007–1.842 copies/cell; interquartile range 0.023–0.67 copies/cell). MCPyV DNA loads were significantly higher in patients with multiple OSCCs than in patients with single OSCC (*P* = 0.011, Mann–Whitney *U* test) (Fig. 1).

MCPyV status and DNA load levels for patients with multiple primaries (16 tumours in 6 patients) are shown in Table 4, along with clinical information (sex, age, smoking/alcohol drinking history, oral sites). Patient 1 developed multiple intraoral primaries at 3 sites, one of which was MCPyV-positive, and the DNA load was 0.025 copies/cell. Patients 2, 4, and 5 developed oral cancers at two sites each, one or both of which were MCPyV-positive, with DNA loads of 0.007–0.702 copies/cell. MCPyV was detected from 3 of 4 oral tumours in Patient 3, and 2 of 3 oral tumours in Patient 6, with relatively high DNA loads of 0.136–1.842 copies/cell. All 6 patients with multiple primary OSCCs were never-smokers, and only one was a regular alcohol drinker.

**Table 4:** DNA load in MCPyV-positive patients with multiple primary OSCCs

	Patient no	Sex	Age	Smoking (Brinkman index)	Alcohol	Oral sites	Oral MCPyV	Copies/cell	MCPyV detection rates of each patient
Multiple primary OSCCs	1	M	87	0	None	Cheek mucosa	+	0.025	1/3 tumours
						Tongue	−		
						Lower gingiva	−		
	2	F	86	0	None	Cheek mucosa	+	0.009	2/2 tumours
						Palate	+	0.007	
	3	F	76	0	None	Lower gingiva	+	0.329	3/4 tumours
						Tongue (Lt)	+	0.577	
						Tongue (Rt)	+	1.826	
						Tongue (Lt)	−		
	4	F	82	0	None	Upper gingiva	+	0.702	1/2 tumours
						Lower gingiva	−		
	5	F	80	0	None	Lip	+	0.022	1/2 tumours
						Cheek mucosa	−		
	6	M	37	0	Every day	Lower gingiva	+	1.842	2/3 tumours
						Cheek mucosa	+	0.136	
						Tongue	−		

### Prognosis

**Fig. 2:**
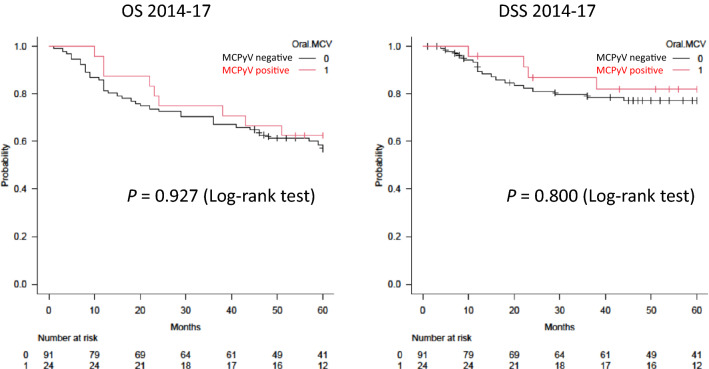
Kaplan–Meier curves for overall survival (OS) and disease-specific survival (DSS) by oral MCPyV DNA status**.** No significant difference in OS or DSS is apparent between oral MCPyV-positive and -negative patients

**Fig. 3:**
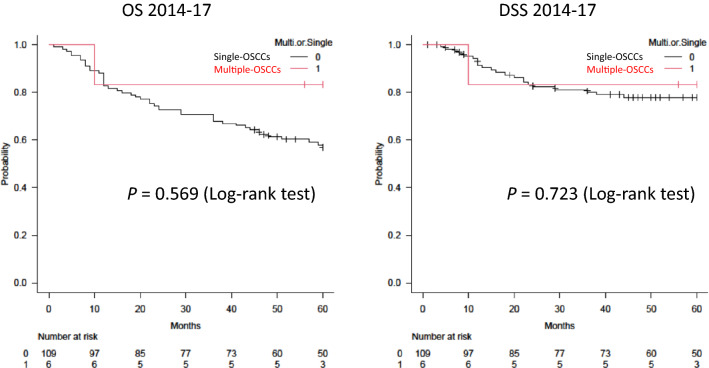
Kaplan–Meier curves for overall survival (OS) and disease-specific survival (DSS) for patients with single and multiple primary OSCCs. No significant difference in OS or DSS is seen between patients with single and multiple primary OSCCs

No significant differences in survival rates (OS or DSS) were identified between groups (MCPyV-positive versus -negative group, and single-OSCC versus multiple-OSCC group) (Figs. 2, 3).

## Discussion

In 2012, the National Institutes of Health in the United States initiated an analysis of the human microbiome as a national project. As a result, microbiomes in the oral cavity, gastrointestinal tract, vagina, nasal cavity, and ears were revealed to interact with the whole body, activating immunity, and showing associations with cancer and immunological diseases [24, 25].

Oral cancer patients often show multiple cancers in the oral cavity and other organs, such as the upper gastrointestinal tract. This phenomenon is explained by the concept of “field cancerization” proposed by Slaughter, et al. in 1953 [12], but the risk factors may involve not only smoking and alcohol drinking, but also the oral microbiome and virome [13–15].

The incidence of multiple oral cancers varies from country to country. Qaisi et al. reported 20/1478 cases (1.4%) with triple cancer or more in Baltimore in the United States [7], while Kawasaki et al. in Japan reported 20/261 cases (7.7%) [8]. In both reports, gingival cancer accounted for 45% of multiple oral cancer cases [7, 8]. In addition, Friedrich in Germany reported 49/649 cases (7.5%) with triple cancer or more [9], and Adel et al. reported 436/1836 cases (23.7%) from a betel quid-chewing endemic area of Egypt [10]. In the present study, 6 of 115 patients (5.2%) developed multiple oral cancers. Although the reports from Qaisi et al., Kawasaki et al., and Friedrich did not mention the characteristics of the multiple oral cancers [7–9], we identified oral MCPyV infection (including consideration of both infection rate and DNA load) as a risk factor for multiple primary OSCCs in a Japanese population. This evidence is further supported by the fact that all 6 patients with multiple primary OSCCs were never-smokers and only one was a regular alcohol drinker.

MCPyV is a novel virus identified in 2008 from a type of cutaneous cancer [26]. Seroprevalence of MCPyV in Europe and Asia have shown that MCPyV infection is very common (approximately 46–88%) among healthy adults and is generally acquired asymptomatically via saliva and/or skin during early childhood, and was about 50% in Japanese adults [27–34]. The virus was shown to be present in the oral cavity as a "wild type", and detected in the oral mucosa, saliva, and gastrointestinal tract, including the large intestine and oesophagus, and has been reported to acquire oncogenic potential due to tumour-specific mutations [17]. HPyV6 and HPyV7, which were both identified in 2010, have also been associated with some tumours, suggesting their roles as oncoviruses [35].

In previous reports on MCPyV expression using FFPE samples of OSCC, Tanio et al. in Japan reported that 7 of 176 OSCC cases (4.0%) (tongue: 2/60 cases, 3.33%; gingiva: 4/52 cases, 7.7%; oral floor: 1/19 cases, 5.3%; other sites: 0/45 cases, 0%) were MCPyV DNA-positive [36], and Hamiter et al. in the United States reported 6 of 21 cases of tongue SCC (28.6%) as being positive [37]. In addition, Saláková et al. in the Czech Republic reported that 40 of 112 malignant tonsillar cases (35.7%) and 11 of 108 non-malignant tonsillar cases (10.2%) were positive for MCPyV DNA [38], Mohebbi et al. in Iran reported that 8 of 50 HNSCC cases (16.0%; including 30 cases of oral cancer) and 1 of 50 non-malignant tissue cases (2.0%) were positive [39], and Muñoz et al. in Chile reported that 15 of 120 OSCC and oropharyngeal SCC cases (12.5%) and 1 of 54 non-malignant tissue cases (1.8%) were positive [40]. From these results, the prevalence of MCPyV in OSCC appears to be 4–35%, significantly higher than that in non-malignant tissue (1.8–10.2%), although some variability of results was seen. In this study, the MCPyV-positive rate in the single-OSCC group was 16.5% (18/109 tumours), compared to 62.5% (10/16 tumours) in the multiple-OSCC group, and the MCPyV DNA-positive rate was significantly higher in the multiple-OSCC group (*P* < 0.001). Furthermore, MCPyV DNA load was significantly higher in the multiple-OSCC group than in the single group (*P* < 0.05). Such results suggest that long-term MCPyV infection may induce various gene mutations and epigenetic abnormalities that result in multiple OSCCs. However, the results of this study alone are insufficient to determine whether MCPyV exists as a bystander or as an oncovirus for multiple OSCCs. Our laboratory previously performed immunohistochemical analyses with CM2B4 monoclonal antibody to examine the expression of MCPyV LT antigen and evaluate its localization in non-small cell lung cancer [20]. Specific strong, diffuse nuclear signals were observed in cancer cells of lung SCC tissue. In the future, we intend to clarify the mechanisms through which MCPyV acts as an oncovirus in OSCC, determine viral DNA polymorphisms by sequencing the *large T* (*LT*) and *small T* (*ST*) gene regions of MCPyV, and accumulate evidence of infection with acquired pathogenicity (tumorigenicity).

No significant difference in prognosis was found between MCPyV-positive and -negative groups, or between single-OSCC and multiple-OSCC groups. This study used OSCC samples from 115 patients over the 4-year period from 2014 to the end of 2017. Although the sample size was sufficient, the number of cases in the multiple-OSCC group was limited (6/115 patients, 5.2%). Therefore, although no significant difference in survival rate was detected, survival rates may differ in the MCPyV-positive and multiple-OSCC groups, and a large-scale case–control study is therefore warranted in the future. MCPyV, which infects the oral cavity, may prove similar to HPV, which infects the oropharynx and is related to the onset and pathology of cancer, but MCPyV may be an oncovirus leading to conditions with a relatively favourable prognosis.

As a result of a phylogenetic analysis of the polyomavirus group, our research group previously discovered and reported genotypes unique to the Japanese population [21, 22, 41, 42], and the presence of HPV and MCPyV co-infection in Japanese cases of cervical cancer [43]. By searching for the presence of not only MCPyV, but also other viruses using these samples, we revealed the possibility that oral virus co-infections (viral networks) may play a role in tumorigenesis, and such results may lead to the creation of biomarkers.

Finally, this study evaluated the effect of MCPyV alone on OSCCs, but we already have preliminary data on viral co-infection from DNA extracted from not only primary tumours, but also metastatic lung and other organ cancer tissues from the same OSCC patients (data not shown). With further advances in these kinds of studies, we expect to be able to elucidate the effects of the oral virus network on field cancerization.

## Conclusions

MCPyV was observed more frequently and DNA loads were significantly higher in patients with multiple primary OSCCs compared to those with single primary OSCC. MCPyV may thus play some role as an oncovirus in multiple primary OSCCs.

## Data Availability

The data that support the findings of this study are available on request from the corresponding author. The data are not publicly available due to privacy or ethical restrictions.

## Abbreviations

OSCC: Oral squamous cell carcinoma
MCPyV: Merkel cell polyomavirus
bp: Base pairs
HPV: Human papillomavirus
HPyV6: Human polyomavirus type 6
HPyV7: Human polyomavirus type 7
FFPE: Formalin-fixed paraffin-embedded
PCR: Polymerase chain reaction
qPCR: Quantitative real-time polymerase chain reaction
OS: Overall survival
DSS: Disease-specific survival
